# T-RECs: rapid and large-scale detection of recombination events among different evolutionary lineages of viral genomes

**DOI:** 10.1186/s12859-016-1420-z

**Published:** 2017-01-05

**Authors:** Michail Tsimpidis, Georgios Bachoumis, Kalliopi Mimouli, Zaharoula Kyriakopoulou, David L. Robertson, Panayotis Markoulatos, Grigoris D. Amoutzias

**Affiliations:** 1 Department of Biochemistry and Biotechnology, Bioinformatics Laboratory, University of Thessaly, Larisa, Greece; 2 Department of Biochemistry and Biotechnology, Molecular Virology Laboratory, University of Thessaly, Larisa, Greece; 3 Faculty of Life Sciences, University of Manchester, Manchester, UK

**Keywords:** Virus, Recombination, Pairwise alignment, Similarity plot, Graphical tool, Norovirus

## Abstract

**Background:**

Many computational tools that detect recombination in viruses are not adapted for the ongoing genomic revolution. A computational tool is needed, that will rapidly scan hundreds/thousands of genomes or sequence fragments and detect candidate recombination events that may later be further analyzed with more sensitive and specialized methods.

**Results:**

T-RECs, a Windows based graphical tool, employs pairwise alignment of sliding windows and can perform (i) genotyping, (ii) clustering of new genomes, (iii) detect recent recombination events among different evolutionary lineages, (iv) manual inspection of detected recombination events by similarity plots and (v) annotation of genomic regions.

**Conclusions:**

T-RECs is very effective, as demonstrated by an analysis of 555 Norovirus complete genomes and 2500 sequence fragments, where a recombination hotspot was identified at the ORF1-ORF2 junction.

## Background

Recombination in viruses is the process that creates chimeric molecules from parental genomes of different origin [1]. Recombination, coupled with point mutations are the main mechanisms of mutation contributing to virus evolution [1, 2]. As demonstrated in Genetic Algorithms, point mutations allow some investigation of the evolutionary fitness landscape, whereas recombination leads to “jumps” within this landscape and exploration of other regions [3]. Furthermore, recombination has been identified as a mechanism of combining advantageous properties from various genomes into a new one, of eradicating deleterious mutations, i.e. acting against Muller’s Ratchet. Recombination can thus be important in the emergence of drug resistance, or for evasion from the immune system [1, 4, 5]. In addition, it breaks the assumption in molecular phylogenetic analyses of a single evolutionary history [6]. Therefore, it is paramount to be able to detect recombination events and correlate them with new properties, or new outbreaks.

Many bioinformatics tools have been developed within the last 20 years that detect recombination events [6–8], and listed at http://bioinf.man.ac.uk/robertson/recombination/programs.shtml. These tools are based on four major categories of methods, such as pairwise sequence comparison, phylogenetics, population genetics, patterns of sites. Evaluations based on simulated and empirical data indicate that an optimal strategy requires implementing more than one method [9, 10]. Nevertheless, the current popular methods and many others are not optimized for analyzing hundreds or even thousands of complete viral genomes in one step (click and forget) and/or in a graphical environment that is user-friendly.

This problem will soon be exacerbated by the recent advances in sequencing technologies, where several popular platforms like Illumina, Ion Proton and Oxford Nanopore allow the rapid sequencing of complete viral genomes [11, 12]. Given the fact that viral genomes in general are of small size, do not contain many and large repeat regions, or that their gene structure is compact and remains stable within a virus species, an explosion of sequenced viral whole genomes is already happening.

Our motivation was to develop a computational pre-filtering tool, named T-RECs (**T**ool for **REC**ombination**s**) that is based on the BLASTN heuristic local pairwise alignment method with sliding windows (http://www.ncbi.nlm.nih.gov/books/NBK1762/). The tool expands on the basic principles of the NCBI genotyping web tool [13] and the SWeBlast perl scripts [14], but as a locally installed program in a Microsoft Windows environment, with a user-friendly graphical interface that allows large-scale analyses and visualization. It rapidly scans hundreds or even thousands of query genomes or even sequence fragments, allows genotyping based on a user-defined sequence database and detects candidate recombination events among members of different evolutionary groups e.g. organisms, genogroups, genotypes etc. The detected candidate events may later be further analyzed (within T-RECs) with similarity plots that integrate the Blast results and even genomic/functional annotations. T-RECs also allows the clustering with Usearch/Uclust [15] of sequences based on a user-defined similarity threshold and thus removes redundancy and simplifies large-scale analyses with many highly similar sequences. In addition, the tool allows for certain regions of a sequence to be selected and uploaded to NCBI Blast or saved. After this pre-filtering step with T-RECs, identified targets may be later analyzed with other more specialized methods/tools.

## Implementation

The software is implemented in Visual Basic and has been tested for Windows 7, 8, 8.1 and 10 operating systems. The executable files for Blastn, Makeblastdb [16] and Muscle (http://drive5.com/muscle) [17] are integrated within the software, but the user is required to manually download the Usearch executable file, so as to integrate sequence clustering and reduction of redundancy (http://www.drive5.com/usearch/) [15] (see accompanying installation videos). The analysis of the 555 Norovirus genomes was performed on a regular laptop (intel core i7) within 3.5 h and required less than 3GB of RAM. Due to the inherent limitations of Visual Basic, the largest sequence that may be analyzed is two gigabases (https://msdn.microsoft.com/en-us/library/thwcx436.aspx).

### The Algorithm

#### The basic steps

Several basic steps are required for the analysis: First, the user uploads the input data that consist of i) a FASTA file of query sequence/s ii) another FASTA file that contains annotated sequences that will be automatically converted within T-RECs with the makeblastdb Blast tool to the BLASTN database iii) an annotation file of viral clusters/groups that is in tab-delimited format, where each row contains the sequence ID and the designated phylogenetic group, for all the sequences of the two FASTA files (query and database) and iv) optionally, a functional annotation file that designates potential regions of interest in the analyzed genomes, such as ORF boundaries, or sites of interest, e.g. post-translational events, sites that confer drug-resistance etc (see the basic analysis video that accompanies the software).

In the second step, the software performs BLASTN on the sequences of the query file with default or user-adapted BLASTN and sliding window parameters. Potential recombination events are identified with the detected acceptor and donor sequences and visualized in a graphical window.

**Fig. 1:**
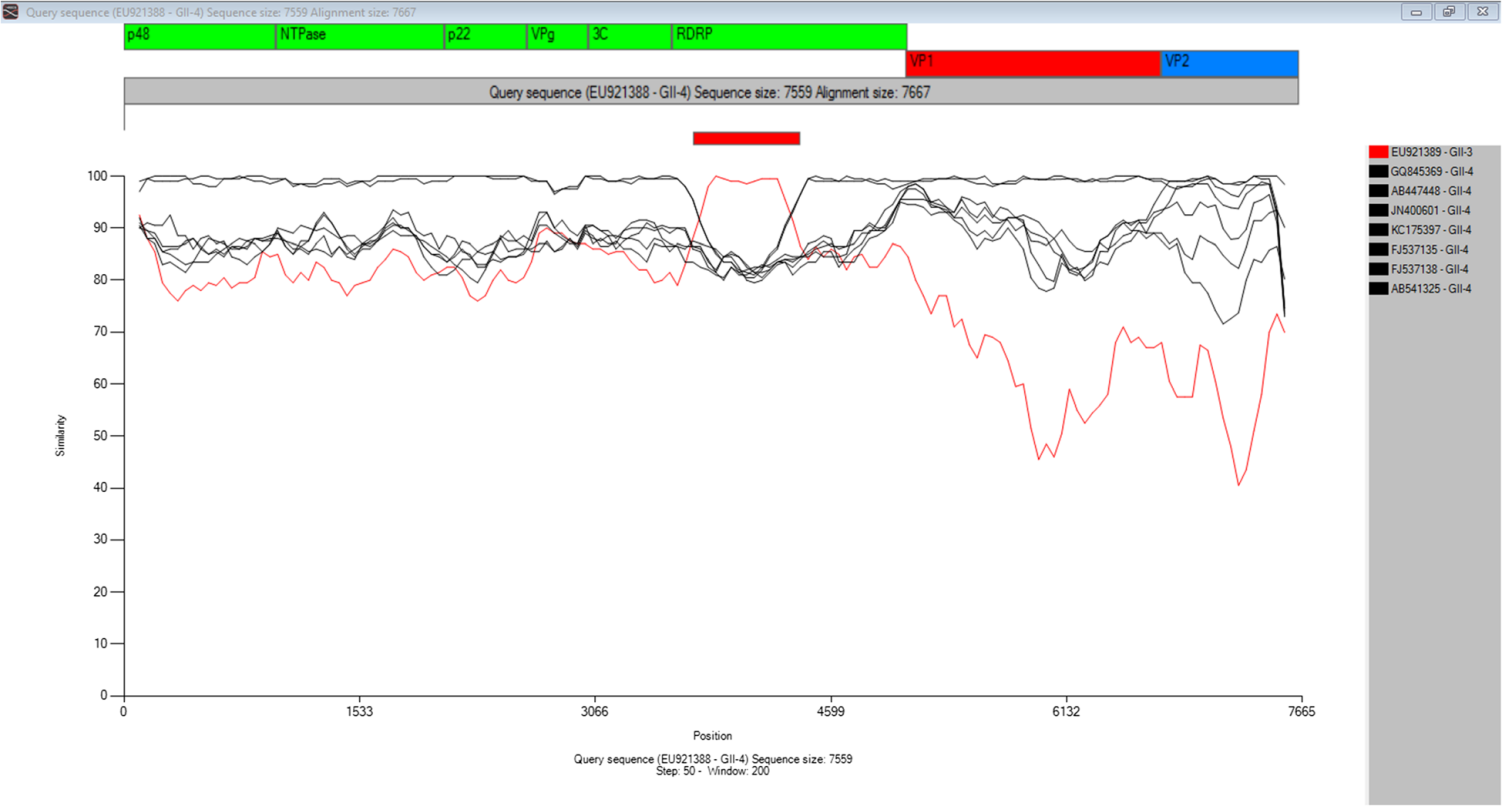
Integrated view of a T-RECs recombination result with similarity plot and ORF annotation that shows a clear recombination event, detected by pairwise alignment of sliding windows and manually verified by similarity plot. This recombination event has been proposed to be an artificial recombinant

In the third step, each potential recombination event can be further analyzed with a similarity plot within T-RECs. The plot may integrate the previous BLAST results as well as functional annotation (see Fig. 1). Within that similarity plot, the user may also opt to select a region of interest from the query sequence and upload it to NCBI Blast. For the similarity plot, the user may also include (apart from the query and the heterologous best blast hit/s) one or more database sequences of the same evolutionary group as the query. In addition, the user may perform multiple alignment of these sequences with Muscle (within T-RECs). The similarity plot parameters, such as sliding window size and increment size, treatment of gaps, and colour of sequences within the graph are user adaptable.

**Fig. 2:**
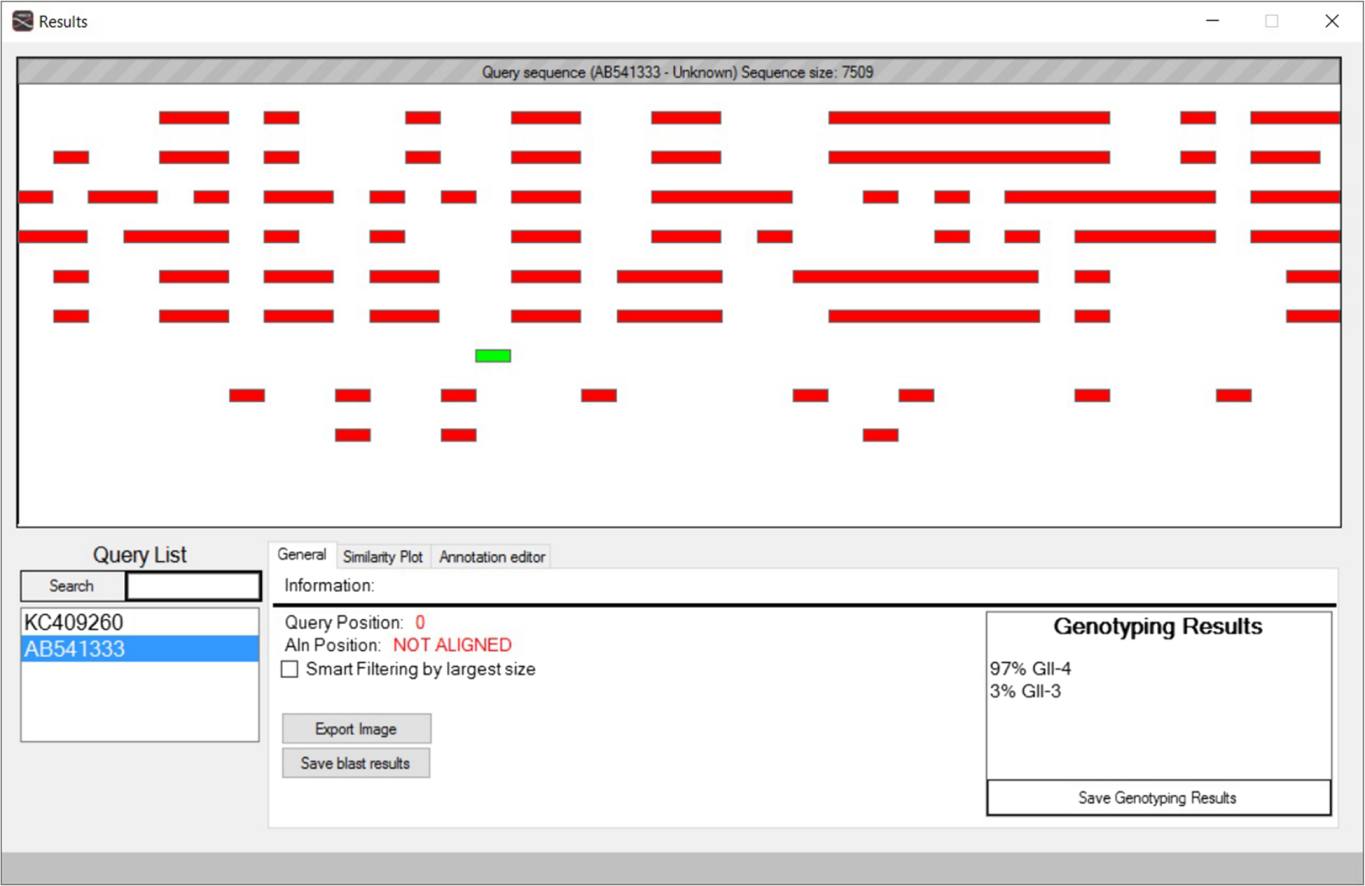
View of genotyping with T-RECs. The grey bar on top is the query sequence, whereas the red rectangles are the best blast sequence fragments of database sequences

As an optional initial step, the user may perform genotyping of the query sequences by using a database with sequences of defined genotypes (Fig. 2). Once the genotypes of the query sequences are identified, they are saved and used for the downstream recombination analyses. For example, in Picornaviruses or Noroviruses, genotyping is based on the VP1 capsid region. The user may create a genotyping database, by selecting only VP1 sequences from the various phylogenetic groups. Based on this first genotyping step, the query sequences may later be scanned for recombination events against another database of complete genomes (of known genotypes).

**Fig. 3:**
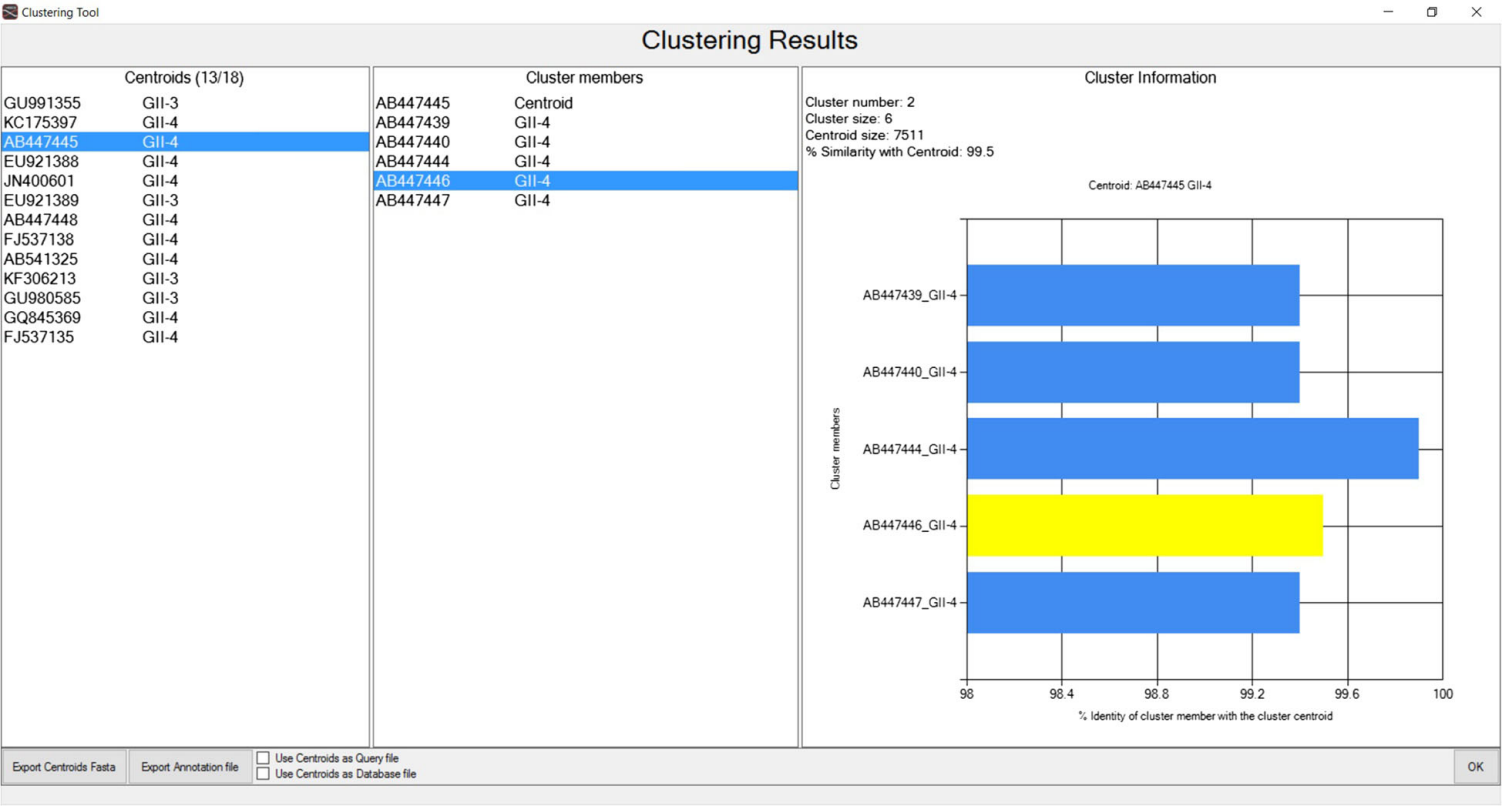
Clustering of sequences, based on nucleotide identity threshold, with Uclust. Centroids denote the representative sequence from each cluster, as selected by Uclust

As an optional extra step, the user may remove redundancy of the query sequences based on a user-defined nucleotide identity threshold, with the help of the Usearch/Uclust software and more specifically of the Uclust command (Fig. 3), after it has been embedded within T-RECs. Also, if the user does not know the genotypes of the sequences, genotype names may be assigned by clustering the sequences with a user-defined similarity threshold and using the cluster name as a genotype.

Apart from a user-guide, all steps of an analysis are also demonstrated in videos that accompany the software.

#### How the algorithm works

T-RECs is based on the principle of sliding windows. The tool breaks each query sequence into sequence fragments with a sliding window of user-defined size and increment. We believe that the optimal window size may be different for the various viruses and should be selected by a user that better understands the evolutionary nature of the virus under investigation. Of note, small fragments are not suggested, since they may be susceptible to convergent evolution effects. Each of those fragments is blasted against the database. If there is no recombination event, then one expects all the fragments of the query sequence to have as their best blast hit another database sequence of the same phylogenetic group. If at some point a certain fragment has as best blast hit a database sequence of another phylogenetic group, then a stringent criterion needs to be fulfilled in order to designate this as a recombination event. The nucleotide identity of the heterologous best blast hit must be 5% (default parameter - can be adjusted by the user, depending on the virus under investigation and sliding window size) higher than the identity of the second best blast hit of the same evolutionary group as that of the query. For example, the query sequence belongs to genotype A and all its sequence fragments have as best blast hit other database sequences of genotype A, apart from a region (e.g from 1000 to 2000) that has as best blast hit a database sequence of genotype B with 99% nucleotide identity. For that same region (e.g from 1000 to 2000), the best blast hit of the same genotype A needs to have a nucleotide identity of 94% or less, in order for genotype B to be recognized as a potential donor. If the nucleotide identity difference is less than the user-defined cutoff, then it is not detected as a recombination event. This cutoff of nucleotide identity percent difference is user-adjustable, depending on the divergence within the groups. If two or more heterologous groups are found as equal best blast hits and the nucleotide identity difference cutoff is satisfied, then all of them are reported as potential donors.

The current algorithm is designed for identifying recent recombination events. For example, if a recombination event happened at some time point and this led to a cluster/group of circulating recombinants, e.g., where two or more of the new recombined genomes have been sampled, then the tool will fail to identify the event. This is because each of the two sequences will have as best blast hit the other recombined sequence. One solution to this problem is prior to the T-RECs recombination analysis, to cluster all the sequences (with Uclust embedded) with a high nucleotide identity cutoff (e.g. >98%) and retain only one representative for further analysis with T-RECs. Obviously, this analysis step may lead to different representatives and thus hide/reveal some older recombination events. In such a case, the user will have to re-analyze the results with various reasonable nucleotide identity cutoffs.

## Results

### T-RECs implementation on 555 norovirus complete genomes

In order to assess the functionality of the software, we analyzed 555 complete human Norovirus genomes, downloaded from Genbank (July 2014) as a proof of principle [18]. A custom Perl script (included in Additional file 1) was developed to automatically extract the GenBank annotation concerning the evolutionary group that the sequences belong to. Next, manual inspection of the automatically extracted data was needed, due to the diverse ways a sequence could be annotated. Also, genome annotation that defines the boundaries of the three ORFs and their eight peptides was extracted, whenever available. ORF1 encodes a polypeptide that matures in 6 peptides (p48, NTPase, p22, VPg, 3C, RNApol,), whereas ORFs 2 and 3 encode two capsid proteins (VP1 and VP2 respectively). Genogroup and genotype classification is based on the phylogeny of the ORF2 (VP1 region). Therefore, whenever the Genbank annotation was not clarifying the genogroup/genotype of the investigated complete genome, we assigned it based on a phylogenetic analysis of ORF2. Multiple DNA alignments (based on aminoacid multiple alignments of the corresponding peptides) were performed for each of the 8 peptide-regions with Muscle (within the Seaview software), whereas the corresponding phylogenetic trees were generated with Maximum Likelihood by PhyML [17, 19, 20]. Tree branch support was estimated with approximate likelihood ratio test (aLRT). The selection of the various evolutionary models for each region (Jukes-Cantor, GTR, K80, TrNef) was based on jModelTest [21]. Trees and their annotations were visualized in Treedyn [22]. All the above datasets are available in Additional file 1. We also compared the performance of T-RECs with the well established and very frequently used Recombination Detection Program (RDP - version 4), that is based on aligned sequences and also incorporates several other published methods [23, 24]. For analysis with RDP(v4), the alignments of the 8 regions were concatenated into one.

Previous small and medium scale published analyses have repeatedly indicated high recombination rates among genogroups and genotypes of this virus, especially at the ORF1-ORF2 junction [25–34]. In addition, the Recombination Analysis Tool (RAT) was specifically evaluated on Norovirus sequences [31]. In our analysis, RDP(v4) detected eleven inter-group recombined sequences that had minor donor fragments larger than 200 nt (see excel table and Similarity plots in the Additional file 1 subdirectory: 555_Norovirus_genomes). Manual inspection revealed that seven of those eleven sequences had clear recombination events, one sequence was the only representative of its evolutionary group (GII-13 capsid region) and had no other highly similar sequences of the capsid region in our sequence database and three events were old. By the term “old”, we mean recombination events that are shared by two or more highly similar sequences of the same evolutionary group. The same 555 complete genome sequences were scanned with T-RECs (sliding window size of 200 nt, step of 50 nt). The 555 genomes were used both as queries and database. This time, the seven sequences that were clearly identified as recombined by RDP(v4) were also identified by T-RECs. Two of the three old events detected by RDP(v4) were missed by T-RECs, one old event detected by RDP(v4) was also detected by T-RECs, one T-RECs event was a potential false positive and there was an event detected by T-RECS that was also detected by RDP(v4), but with a minor donor fragment of 160 nt. Finally, the single representative sequence (GII-12/13) was identified correctly by RDP(v4) as a recombinant ORF1 and was also detected by T-RECs, but with the recombination occurring at the capsid region (due to lack of other representatives of GII-13 in the database). All these detected recombination events were validated by the phylogenetic trees and by similarity plots within T-RECS and double-checked with the Simplot software (phylogenetic trees and similarity plots of the potential recombination events are stored in the Additional file 1: subdirectory 555_Norovirus_genomes). The majority (nine) of the recombination breakpoints were situated at the ORF1-ORF2 junction region. In addition, there was one recombination event at the ORF2-ORF3 junction, another event where the N-terminal half of the RNA-dependent RNA Polymerase region came from another group (this event is suspected to be an artificial recombinant [26]) and another event involving the section spanning p22 and VPg.

### T-RECs implementation on 2538 norovirus sequence fragments

In addition, we performed a T-RECs analysis with 2538 phylogeneticaly annotated sequence fragments of size >300 nt, downloaded from Genbank [18]. The 2538 sequences were used both as queries and database. One of the great advantages of the software is that it can analyze sequence fragments of varying length, without any prior data manipulation (as long as the genotype/genogroup is known). T-RECs identified 56 potential recombination events (with a sliding window of 200 nt and an increment of 50 nt). Nevertheless, manual inspection and Simplot analysis revealed that the majority could either be artifacts due to potential mis-annotation in Genbank or they were not clearly defined events (e.g. the donor was unknown or interpretation of the plot was not feasible). We considered a detected recombination as potential mis-annotation when a whole sequence fragment of a certain genotype was clearly giving as best blast hit a sequence of another genotype, throughout its whole length and with no visible evidence of recombination within that fragment. These cases were not investigated any further. We identified eight results, where the recombination point was clearly seen on the query fragment, seven of which were situated at the ORF1-ORF2 junction (see Additional file 1) and one situated at the middle of the RNA-dependent RNA Polymerase region. All the similarity plots of the detected recombinations (clear and dubious) are available for manual inspection in the Additional file 1 subdirectory: 2538_Norovirus_fragments:Simplots. Nevertheless, a bias exists, because most sequenced fragments are designed to contain ORF2, thus, the most unbiased picture is provided from whole genome analyses.

## Discussion

Numerous recombination detection software have been developed within the last 20 years, based on four major categories of methods, i) pairwise sequence comparison, ii) phylogenetics, iii) population genetics and iv) patterns of sites. Methods that are based on pairwise local alignment of sliding windows hold the potential to analyze in a simple and rapid manner huge amounts of genomic data. Therefore, they are ideal as the first step in a multi-method search strategy. An available computational tool that falls in this specific category is the web-based NCBI genotyping tool, that is based on BLAST [13]. The user may also define a custom sequence database and results may further be analyzed/visualized with similarity plots. Yet, this online tool cannot analyze more than one submitted query sequence at a time. Another computational tool of the same category is SWE-Blast, a command line tool that breaks the query sequence in fragments with a sliding window, submits each fragment to NCBI blast and then parses the results in text files [14].

Incongruence of phylogenetic trees and similarity plots [35] are among the most popular approaches. A common problem of detecting recombination events with phylogenetic analyses is that it is necessary to define the boundaries of the genomic regions to be analyzed. If the recombination breakpoint is in the middle of one of those regions, then this sequence fragment may cluster incorrectly with other sequence fragments in the tree, and usually with low bootstrap support. One way to circumvent this problem is to use the Bootscan method [36], but, if the number and length of sequences is too large, then the analysis becomes problematic in terms of time and complexity. In addition, bootstrap values may be low, either due to other sequences not clustering reliably or due to lack of phylogenetic signal in a region. Similarity plots also allow for detection of the recombination breakpoint/s, but only on a small number of sequences. Otherwise, visualization, manual inspection and interpretation of results from an analysis of many sequences becomes very complicated.

RDP(v4) is a very popular software suite that combines many diverse recombination detection methods, but requires the sequences to be aligned [23, 24]. Such an alignment step may become very time and labor costly, especially for an analysis of thousands of genomes, because the DNA alignment would need to be based on protein alignments of each ORF. Therefore, the current popular methods and many others are not optimized for analyzing hundreds or even thousands of complete viral genomes in one step (click and forget) and/or in a graphical environment that is user-friendly.

T-RECs is a Visual Basic graphical tool powered by blast, muscle and Usearch/Uclust that rapidly scans hundreds or even thousands of viral genomes or sequence fragments for recombination events. It is based on pairwise Blastn alignment of sliding windows of query sequences against a user-defined sequence database. Query sequences may be further genotyped or clustered based on sequence identity. Recombination hits may also be visually inspected with Similarity Plots within T-RECs, or submitted to the NCBI blast website for further analysis. T-RECs is recommended as a high-throughput pre-filtering tool for recombination events that would be further analyzed by other more specialized software, optimized for small/medium scale studies, such as Simplot, RDP(v4), HyPhy, Bootscan, Maxchi, Chimaera, 3SEQ, VISRD, LARD, Siscan, etc. [10, 23, 24, 35–43]. T-RECs requires that all sequences have an assigned genotype, in order to identify recombinations. In case that a user does not want to define these genotypes via lengthy phylogenetic pre-analyses, T-RECs simplifies this step by an embedded genotyping tool, although prototype sequences are still needed. Otherwise, another alternative is to cluster the sequences (with the embedded tool) with a user-defined similarity threshold and use the cluster names as genotypes, so as to proceed to detection of recombination events. Although T-RECs is not capable of identifying older recombination events in the first run, there is an option to cluster the sequences with a user-defined similarity threshold and proceed to recombination detection with only one representative sequence from each cluster. Also, we recommend that the selected sliding window size is large enough to avoid confounding factors stemming from convergent evolution (on a certain region) or from differences in evolutionary rate.

T-RECs has been evaluated in this analysis with Norovirus genomes. Also, it has been tested successfully (results not shown) with Picornaviruses. T-RECs has neither been optimized for nor tested in extreme cases of very large viral genomes (in the range of Megabases), nor even in cases of bacterial genomes. Nevertheless, the vast majority of viral genomes (especially RNA viruses) are in the range of a few tens of kilobases [44–46] and are therefore within the limitations and capabilities of T-RECs.

In a way, most of the advantages of the NCBI Genotyping tool, SWeBlast and Simplot are recombined into the T-RECs tool, and further adapted/optimized for this new era of Next-Generation Sequencing.

## Conclusions

T-RECs is a Microsoft Windows based graphical tool that rapidly scans (within hours) hundreds or even thousands of viral genomes or sequence fragments for recombination events and with fairly limited computation resources that are found in common desktops/laptops. It is based on pairwise Blastn alignment of sliding windows of query sequences against a user-defined sequence database. Query sequences may be further genotyped or clustered based on sequence identity. Recombination hits may also be visually inspected with Similarity Plots within T-RECs, or submitted to the NCBI blast website for further analysis. T-RECs is recommended as a high-throughput pre-filtering tool for recombination events that may be found in the vast majority of viruses. Once these events are detected, they would need to be further analyzed by other more specialized software that are optimized for small/medium scale studies.

## Availability and requirements

Project name: T-RECs.

Project homepage: http://bioinf.bio.uth.gr/t-recs.html.

Operating system: Microsoft Windows.

Programming Language: Visual Basic.

Other requirements: USEARCH/UCLUST.

## Additional file

Additional file 1: All Additional files containing Norovirus sequences, alignments, phylogenetic trees Similarity Plots and the PERL script are compressed and can be downloaded from http://bioinf.bio.uth.gr/downloads/T-RECs_Supplementary_files.zip.

## Abbreviations

aLRT: Approximate likelihood ratio test
BLAST: Basic local alignment search tool
NCBI: National Center for Biotechnology Information
ORF: Open reading frame
RDP: Recombination detection program
T-RECs: Tool for RECombinations
